# Recovery from critical illness-induced organ failure: the role of autophagy

**DOI:** 10.1186/s13054-017-1786-y

**Published:** 2017-08-07

**Authors:** Jan Gunst

**Affiliations:** Clinical Division and Laboratory of Intensive Care Medicine, Department of Cellular and Molecular Medicine, KU Leuven University and Hospital, Herestraat 49, 3000 Leuven, Belgium

**Keywords:** Critical illness, Sepsis, Autophagy, Nutrition, Insulin, Multiple organ dysfunction syndrome

## Abstract

Autophagy is a catabolic process by which cells can dispose of damaged content and intracellular microorganisms. Recent evidence implicates autophagy as a crucial repair process necessary to recover from critical illness-induced organ failure. Withholding parenteral nutrition in the acute phase of critical illness activates autophagy and enhances recovery. Several registered drugs have autophagy-stimulating properties, but all lack specificity and none has been investigated in critically ill patients for this purpose.

## Main text

In critical illness, a variety of stressors may induce cellular damage and lead to organ failure. After treatment of the primary condition leading to intensive care unit (ICU) admission and general ICU care, the majority of patients will recover. However, despite maximal care, approximately 10–30% of patients do not swiftly recover and enter a phase of prolonged critical illness, characterized by a prolonged dependency on vital organ support and associated with a high risk of mortality and long-term debilities [1, 2]. The underlying reasons why certain patients quickly recover whereas others remain ICU-dependent are incompletely understood. Despite the severe organ failure, frank necrosis or apoptosis are uncommon, and in patients surviving this condition, (partial) recovery is possible, even when organs with poor regenerative capacity are involved. This suggests that the cells from critically ill patients dispose of repair mechanisms that allow—at least partially—reversal of the organ dysfunction. Recent evidence implicates macroautophagy, hereafter referred to as autophagy, as a crucial cellular repair process that is essential to recover from critical illness-induced organ failure.

**Fig. 1:**
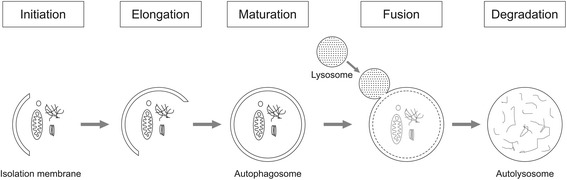
Overview of autophagy. Autophagy initiates with the formation of an isolation membrane (also called phagophore) in the cytoplasm. Isolation membranes elongate to finally form a double-membrane vesicle, the autophagosome. During the elongation process, intracellular cargo (e.g., damaged organelles, protein aggregates, invaded microorganisms) is recruited to the isolation membrane to be engulfed. After maturation of the autophagosome, the vesicle fuses with a lysosome, resulting in formation of an autolysosome, in which the sequestered content is degraded and thereafter recycled to the cytoplasm. The figure is original for this article

Autophagy, literally meaning “self-eating”, is a catabolic process by which intracellular content is delivered to the lysosome via an intermediate organelle, the autophagosome [1]. Autophagosomes are intracellular vesicles formed out of isolation membranes in the cytoplasm, which elongate around its substrate to finally form a vesicle (Fig. 1). Substrates may be recruited to growing autophagosomes by tagging molecules such as ubiquitin. Mature autophagosomes fuse with lysosomes, after which the engulfed content is degraded and recycled. Autophagy can be activated by starvation, exercise, and a variety of stress signals. Nutrients, growth factors and insulin suppress autophagy. Importantly, autophagy is the only process that is able to remove macromolecular damage, such as damaged organelles and potentially toxic protein aggregates, and intracellular microorganisms. As such, it serves an important homeostatic role, which is illustrated by the severe phenotypes that develop when autophagy is selectively and tissue-specifically knocked out in adult animals. Both insufficient and excessive autophagy have been associated with pathology. Whereas autophagy was originally mainly considered as a negative process, recent evidence has identified autophagy activation as adaptive in many disease states, including critical illness-induced vital organ failure and muscle weakness [1–3].

Early observational studies attributed sepsis-induced organ damage to the concomitant appearance of autophagosomes. However, without interfering with the process, causality is unclear. Recently, a considerable number of studies have provided clear evidence of potentially insufficient autophagy activation in relation to the degree of cellular stress. A pioneer study identified hallmarks of insufficient autophagy activation in liver and muscle of prolonged critically ill patients, with accumulation of autophagic substrate in the presence of a reduced number of autophagic vacuoles [1]. Similar observations were performed in a critically ill animal model, in which the degree of autophagy activation inversely correlated with the degree of respective organ dysfunction and mortality risk, which supports the functional relevance of activated autophagy [4]. Likewise, in patients with prolonged critical illness, the degree of muscular autophagy inversely correlated with the incidence of ICU-acquired muscle weakness [2]. Moreover, administration of the autophagy inducer rapamycin to burn-injured critically ill rabbits activated autophagy and protected against organ dysfunction and bone loss [4, 5]. Recently, a considerable number of studies have confirmed a protective role of autophagy against various types of organ failure in critically ill animal models. Indeed, in rodents, active autophagy protected against ischemia-reperfusion injury in various organs as well as against sepsis- or endotoxin-induced pulmonary, renal, cardiac, hepatic, neuronal, and immune dysfunction/injury and against sepsis-induced mortality [6–13].

The necessary activation of autophagy in response to severe stress may be affected by artificial feeding and insulin therapy. Human and animal studies have demonstrated significant autophagy suppression by early parenteral nutrition (PN) in critical illness, which coincided with more organ damage and—in patients—a prolonged dependency on vital organ support [2, 14]. Among the different macronutrients, amino acids especially strongly suppressed autophagy, more than glucose or lipids [14]. Although speculative, this may explain why amino acid administration, and not glucose or lipids, statistically explained the harm evoked by early PN in a multicenter clinical study [15]. Although insulin is a known suppressor of autophagy, tissular glucose overload evoked by severe hyperglycemia may also impair autophagy. Hence, lowering blood glucose with insulin may influence autophagy in both directions and the net effect remains unclear. Whereas a study on human samples found a reduction in autophagic vacuoles following this therapy in liver, other hepatic autophagy markers and muscular autophagy were unaffected [1]. An animal study demonstrated improved autophagy in liver and kidney by preventing severe hyperglycemia with insulin [4]. Importantly, in both human and animal studies, early PN was administered and in this context, maintaining normoglycemia with insulin protected against organ damage [1, 4]. Hence, w﻿ith early PN, the net balance between genesis and removal of cellular damage was in favor of preventing severe hyperglycemia with ﻿insulin, even if the net effect on autophagy remains unclear.

Hence, autophagy activation emerges as a potentially important therapeutic target in critical illness. Withholding PN in the acute phase of critical illness was found to stimulate autophagy and improve organ recovery [2]. However, prolonged starvation may come at a price. In an animal study, feeding-induced suppression of autophagy could be overcome by administration of the autophagy inducer rapamycin, which decreased morbidity [4]. However, rapamycin also has potent immune-suppressive effects, which precludes its widespread use in critically ill patients. Likewise, several other registered drugs have been identified as potential autophagy inducers [3]; however, these lack specificity and none has been investigated in critically ill patients for this purpose. Future research should aim at identifying novel, more specific autophagy inducers that are suitable for study in ICU patients.

## Abbreviations

ICU: Intensive care unit
PN: Parenteral nutrition
